# α-Mangostin extracted from the pericarp of the mangosteen (*Garcinia mangostana *Linn) reduces tumor growth and lymph node metastasis in an immunocompetent xenograft model of metastatic mammary cancer carrying a p53 mutation

**DOI:** 10.1186/1741-7015-9-69

**Published:** 2011-06-03

**Authors:** Masa-Aki Shibata, Munekazu Iinuma, Junji Morimoto, Hitomi Kurose, Kanako Akamatsu, Yasushi Okuno, Yukihiro Akao, Yoshinori Otsuki

**Affiliations:** 1 Laboratory of Anatomy and Histopathology, Faculty of Health Science, Osaka Health Science University, Osaka, Japan; 2 Department of Anatomy and Cell Biology, Division of Life Sciences, Osaka Medical College, Takatsuki, Osaka, Japan; 3 Laboratory of Pharmacognosy, Gifu Pharmaceutical University, Gifu, Japan; 4 Laboratory Animal Center, Osaka Medical College, Osaka, Japan; 5 Department of Systems Bioscience for Drug Discovery, Graduate School of Pharmaceutical Sciences, Kyoto University, Kyoto, Japan; 6 United Graduate School of Drug Discovery and Medical Information Science, Gifu University, Gifu, Japan

## Abstract

**Background:**

The mangosteen fruit has a long history of medicinal use in Chinese and Ayurvedic medicine. Recently, the compound α-mangostin, which is isolated from the pericarp of the fruit, was shown to induce cell death in various types of cancer cells in *in vitro *studies. This led us to investigate the antitumor growth and antimetastatic activities of α-mangostin in an immunocompetent xenograft model of mouse metastatic mammary cancer having a p53 mutation that induces a metastatic spectrum similar to that seen in human breast cancers.

**Methods:**

Mammary tumors, induced by inoculation of BALB/c mice syngeneic with metastatic BJMC3879luc2 cells, were subsequently treated with α-mangostin at 0, 10 and 20 mg/kg/day using mini-osmotic pumps and histopathologically examined. To investigate the mechanisms of antitumor ability by α-mangostin, *in vitro *studies were also conducted.

**Results:**

Not only were *in vivo *survival rates significantly higher in the 20 mg/kg/day α-mangostin group versus controls, but both tumor volume and the multiplicity of lymph node metastases were significantly suppressed. Apoptotic levels were significantly increased in the mammary tumors of mice receiving 20 mg/kg/day and were associated with increased expression of active caspase-3 and -9. Other significant effects noted at this dose level were decreased microvessel density and lower numbers of dilated lymphatic vessels containing intraluminal tumor cells in mammary carcinoma tissues.

*In vitro*, α-mangostin induced mitochondria-mediated apoptosis and G1-phase arrest and S-phase suppression in the cell cycle. Since activation by Akt phosphorylation plays a central role in a variety of oncogenic processes, including cell proliferation, anti-apoptotic cell death, angiogenesis and metastasis, we also investigated alterations in Akt phosphorylation induced by α-mangostin treatment both *in vitro *and *in vivo*. Quantitative analysis and immunohistochemistry showed that α-mangostin significantly decreased the levels of phospho-Akt-threonine 308 (Thr308), but not serine 473 (Ser473), in both mammary carcinoma cell cultures and mammary carcinoma tissues *in vivo*.

**Conclusions:**

Since lymph node involvement is the most important prognostic factor in breast cancer patients, the antimetastatic activity of α-mangostin as detected in mammary cancers carrying a p53 mutation in the present study may have specific clinical applications. In addition, α-mangostin may have chemopreventive benefits and/or prove useful as an adjuvant therapy, or as a complementary alternative medicine in the treatment of breast cancer.

## Background

Breast cancer represents a major health problem in women, with more than 1,000,000 new cases and 370,000 deaths yearly worldwide [1]. Perhaps more worrisome is an apparently increasing incidence of breast cancer among younger women under 40 years of age recently reported in many countries worldwide [2-4]. The lethality of breast cancer is largely due to metastasis, preferentially to the lymph nodes, lungs and bones [5]; in order to delay the progression of breast cancer and prolong patient life, more effective chemopreventive and antimetastatic treatments and less toxic chemotherapeutic agents are desperately required.

**Figure 1:**
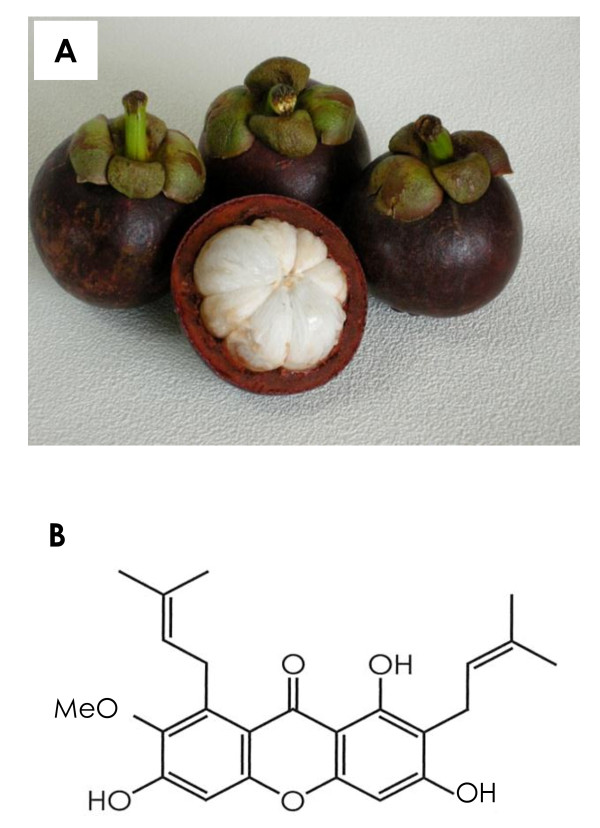
**Gross appearance of α-mangostin and its chemical structure**. **(A) **Gross appearance of mangosteen fruit. The edible endocarp of the mangosteen is snow white and botanically defined as an aril. The circle of wedge-shaped arils contains four to eight segments. **(B) **The chemical structure of α-mangostin; molecular formula, C_24_H_26_O_6_; molecular weight, 410.

The mangosteen (*Garcinia mangostana *Linn) has been dubbed the 'queen of fruit' in its native Thailand. Mangosteens are small (about 4 to 8 cm in diameter) round fruits with a thick, brittle, deep purple spherical outer shell or pericarp. The edible snow white endocarp of the mangosteen is arranged in a circle of wedge-shaped, 4- to 8-segmented arils (Figure 1A). The fruit has a long history of medicinal use in both Chinese and Ayurvedic medicine. For centuries, people in Southeast Asia have used dried mangosteen pericarp for medicinal purposes; it is used as an antiseptic, an anti-inflammatory, an anti-parasitic, an antipyretic, an analgesic, and as a treatment for skin rashes [6].

The compound α-mangostin, which was isolated from the pericarp, has recently been shown to induce cell-cycle arrest and apoptosis in various types of human cancer cells [7-10]. α-Mangostin has additionally been shown to inhibit cell invasion and migration in mammary and prostate cancer cells and is associated with down-regulation of MMP-2 and MMP-9 [11,12]. In one *in vivo *animal cancer model, crude α-mangostin (comprised of 78% α-mangostin and 16% γ-mangostin) administered in the diet significantly suppressed formation of aberrant crypt foci, considered to be a putative preneoplastic lesion, in rat colon carcinogenesis [13]. Furthermore, we have more recently reported that dietary administration of panaxanthone, a compound of approximately 75% to 85% α-mangostin and 5% to 15% γ-mangostin with the sum of both contents > 90%, significantly inhibited both tumor growth and metastasis in a mouse model of mammary cancer [14].

Here, we investigated the antitumor potential of purified α-mangostin, focusing on its antimetastatic ability, in a mouse metastatic mammary cancer model carrying a p53 mutation that demonstrates a metastatic spectrum similar to that seen in human breast cancers [15-17]. In addition, we analyzed the effect of α-mangostin exposure on apoptosis, DNA synthesis, and cell cycle arrest *in vitro *using metastatic mouse mammary carcinoma cells. Since Akt phosphorylation has been shown to participate in cell growth, survival, proliferation, motility, and/or invasion in various cancers, including human breast cancer, we further examined the influence of α-mangostin treatment on Akt phosphorylation both *in vitro *and *in vivo*.

## Methods

### Experimental regimen

Mangosteen (*Garcinia mangostana *Linn) pericarps (Figure 1A) collected in Thailand were dried, ground, and successively extracted in water and 50% ethanol. After freeze-drying the 50% ethanol extract, the resultant dried powder was suspended in water partitioned with ethyl acetate. The ethyl acetate extract was then purified by chromatography on silica gel with the n-hexane-ethyl acetate system and recrystallized to give α-mangostin at > 98% purity. The chemical structure of α-mangostin is shown in Figure 1B. For *in vitro *use, crystallized α-mangostin was dissolved in dimethyl sulphoxide (DMSO), and aliquots of stock 20 mM solution were stored at -20°C.

### Cell lines and animals

The murine BJMC3879 mammary adenocarcinoma cell line was derived from a metastatic focus within a lymph node of an inoculated BALB/c mouse in an earlier study; the cell line continues to show a high metastatic propensity, especially to lymph nodes and lungs [18-20], a trait retained through culture. The BJMC3879luc2 mammary carcinoma cell line used in our investigations was generated by stable transfection of the *luc2 *gene (an improved firefly *luciferase *gene) into the parent BJMC3879 cell line [21]. BJMC3879luc2 cells were maintained in RPMI-1640 medium containing 10% fetal bovine serum with streptomycin/penicillin at 37°C under 5% CO_2_. MDA-MB231, a human mammary carcinoma cell line stably expressing the green fluorescence protein (GFP)[22] was maintained in DMEM or RPMI-1640 medium containing 10% fetal bovine serum. Most of the *in vitro *analyses of caspase, cytochrome *c *release, Bid and cell cycle were conducted using the mouse BJMC3879luc2 cells, but Akt-phosphorylation analysis was performed in both human MDA-MB231 cells *in vitro *and mouse BJMC3879luc2 cells *in vivo*.

Thirty six-week-old female BALB/c mice were used in this study (Japan SLC, Hamamatsu, Japan). The animals were housed five per plastic cage on wood chip bedding with free access to water and food under controlled temperature (21 ± 2°C), humidity (50 ± 10%), and lighting (12-12 hours light-dark cycle) conditions. All animals were held for a one-week acclimatization period before study commencement. This animal experiment was approved by the Animal Experiment Committee of Osaka Medical College. Mice were treated in accordance with the procedures outlined in the Guide for the Care and Use of Laboratory Animals at Osaka Medical College, the Japanese Government Animal Protection and Management Law (No.105) and the Japanese Government Notification on Feeding and Safekeeping of Animals (No.6).

### Cell viability

BJMC3879luc2 and MDA-MB231 cells were grown in RPMI-1640 medium supplemented with 10% (v/v) heat-inactivated fetal bovine serum and 2 mM L-glutamine under an atmosphere of 95% air and 5% CO_2 _at 37°C. These cells were plated into 96-well plates (1 × 10^4 ^cells/well) one day before α-mangostin treatment. They were subsequently incubated for 24 hours with culture medium containing DMSO vehicle alone or with medium containing α-mangostin at various concentrations up to 20 μM. Cell viability was determined using a CellTiter-Bule Cell Viability Assay (Promega Co., Madison, WI, USA). The IC_50 _for each cell line under these conditions was found to be 12 μM α-mangostin in BJMC3879luc2 and 20 μM in MDA-MB231 cells; thus, all *in vitro *studies were performed using exposure to these respective concentrations of α-mangostin for 24 hours.

### Caspase activity and TUNEL assay

BJMC3879luc2 cells were grown in two-well chambered slides and treated with 12 μM α-mangostin for 24 hours. The cells were then fixed in 4% formaldehyde solution in phosphate buffer and terminal deoxynucleotidyl transferase-mediated dUTP-FITC nick end-labeling (TUNEL) staining was performed according to the manufacturer's protocol (Wako Pure Chemical Industries, Osaka, Japan).

BJMC3879luc2 cells were plated into 96-well plates at a concentration of 1 × 10^4 ^cells/well one day before α-mangostin treatment. Cells were treated with 12 μM α-mangostin or DMSO alone for 48 or 72 hours; subsequent cell viability was measured using a CellTiter-Blue Cell Viability Assay (Promega). The activities of caspase-8, caspase-9 and caspase-3 were measured using a luminescent assay kit (Promega). Caspase activity was measured in terms of the luminescent signal produced by caspase cleavage of the corresponding substrate using a Luminoskan Ascent kit (Thermo Electron Co., Helsinki, Finland). Caspase activity levels were then adjusted to account for the corresponding cell viability data as previously reported [16].

### Release of cytochrome *c*

After incubation with either DMSO alone or with 12 μM α-mangostin for 24 hours, both floating and attached BJMC3879luc2 cells were harvested, rinsed once in PBS, re-suspended in cell lysis buffer, incubated for one hour at room temperature, and centrifuged at 1000 × g for 15 minutes. The resultant supernatant was diluted at least five-fold. Supernatants containing the cytosolic fraction were collected separately and the protein concentrations were determined. To evaluate cytochrome *c *release into the cytosol, cytochrome *c *was measured using a Cytochrome *c *ELISA kit (R&D Systems, Inc, Minneapolis, MN, USA).

### Caspase inhibitor experiment

BJMC3879luc2 cells were treated for 24 hours with either 10 μM or 100 μM of the following caspase inhibitors: z (N-benzyloxycarbonyl)-VAD-fmk (fluoromethyl ketone) against broad-spectrum caspases; Ac (acetyl)-DNLD-CHO (aldehyde) against caspase-3; z-IETD-fmk against caspase-8; and z-LEHD-fmk against caspase-9. With the exception of the caspase-3 inhibitor, which was purchased from Peptide Institute, Inc., Osaka, Japan, these caspase inhibitors were purchased from MBL, Inc. Nagoya, Japan. Although DEVD has generally been used as a caspase-3 inhibitor, it has recently been demonstrated as non-specific to caspase-3 [23,24]; in the present experiment, we therefore decided to use Ac-DNLD-CHO to inhibit caspase-3. Two hours after inhibitor treatments, cells were exposed to 12 μM α-mangostin and cell viability was measured 24 hours later using a fluorescent assay kit (CellTiter-Blue Cell Viability Assay, Promega).

### Cell-cycle distribution

Flow cytometric analysis was conducted on trypsinized BJMC3879luc2 cell suspensions that were harvested after 24 hours exposure to 12 μM α-mangostin and fixed in cold 70% ethanol. The cells were stained with a 50 μg/ml propidium iodide solution containing 100 μg/ml RNase A for 30 minutes at 37°C and then placed on ice just prior to flow cytometric analysis (EPICS Elite ESP; Coulter Co., Miami, FL, USA). The percentage of cells in each phase of the cell cycle was determined using a Multicycle Cell Cycle Analysis program (Coulter).

### Western blotting

Total protein was extracted from whole cell lysates of BJMC3879luc2 cells and MDA-MB231 cells treated with DMSO (control) or α-mangostin according to the IC_50 _data previously stated. Total protein (40 μg) was fractionated on 14% Tris-glycine gels under reducing conditions and transferred to nitrocellulose membranes. The membranes were incubated with primary antibodies for the following proteins: Bid, total Akt, phospho-Akt-Thr308, phospho-Akt-Ser473, and β-actin. Membranes were then incubated with the corresponding secondary antibodies conjugated with horseradish peroxidase (HRP). All antibodies were purchased from Santa Cruz Biotechnology (Santa Cruz, CA, USA), with the exception of the antibodies for Bid (R&D Systems) and phospho-Akt-Ser473 (Cell Signaling Technology, Danvers, MA, USA). Antibody binding was subsequently visualized by exposure to an enhancing chemiluminescence reagent (Amersham ECL; GE Healthcare UK Ltd., Buckinghamshire, UK). Blots were visualized using a LAS-3000 image analyzer (Fujifilm, Co., Tokyo, Japan).

### Measurement of Akt phosphorylation

MDA-MB231 cells were treated with 20 μM α-mangostin or DMSO (vehicle control) for up to six hours. Protein was extracted using cell lysis buffer containing protease and phosphatase inhibitor cocktail. Total Akt, Akt phosphorylated-threonine 308 (phospho-Akt-Thr308) and Akt phosphorylated-serine 473 (phospho-Akt-Ser473) were measured with phosphorylation assay kits (AlphaScreen SureFire for Akt signaling and GAPDH, Perkin Elmer, Waltham, MA, USA) using a multilabel plate reader (model EnSpire™ Alpha, Perkin Elmer). Data were corrected against glyceraldehyde-3-phosphate dehydrogenase (GAPDH) values and expressed as mean ± SD.

### *In vivo *study of α-mangostin in a metastatic mammary cancer model

Two dosages of α-mangostin - 10 and 20 mg/kg/day - were selected for the *in vivo *studies in mice based on the results of preliminary investigations. In brief, no differences in body or organ weights were found in mice on a four-week toxicity study of crude α-mangostin administered 0, 4, 20, 40 and 120 mg/kg by gavage. The study demonstrated that mice treated with more than 20 mg/kg/day showed significant increases in NK activity [25]; therefore, since 20 mg/kg/day appears to be the highest concentration that shows no harmful effect, we chose 20 mg/kg as the dose to use in the present study.

It was difficult and expensive to obtain large amounts of the pure α-mangostin. Rather than subject the mice to the stress of daily gavage as well as to minimize unwanted loss of an invaluable test agent, α-mangostin was continuously administered via subcutaneously implanted mini-osmotic pumps (Alzet model 2002, Durect Co., Cupertino, CA, USA) that were calibrated to release 0.5 μl of solution per hour. α-Mangostin solutions (15 mg/ml and 30 mg/ml) in a DMSO/100% ethanol (1:3, v/v) vehicle were prepared. Control mice received the DMSO/100% ethanol vehicle alone. Since the pumps were calibrated to release for 14 days, they were replaced every other week.

BJMC3879luc2 cells, at a concentration of 2.5 × 10^6 ^cells/0.3 ml in phosphate-buffered saline, were subcutaneously inoculated into the right inguinal region of 30 female BALB/c mice. Three weeks later, when tumors had reached approximately 0.4-0.6 cm in diameter, mice were exposed to 0, 10 or 20 mg/kg/day α-mangostin via the mini-osmotic pumps for six weeks. Individual body weights were recorded weekly. Each mammary tumor was also measured weekly using digital calipers, and tumor volumes were calculated using the formula of maximum diameter × (minimum diameter)^2 ^× 0.4 [26]. All surviving mice were euthanized with isoflurane anesthesia after week six. One hour prior to euthanasia, all animals were intraperitoneally injected with 50 mg/kg 5-bromo-2'-deoxyuridine (BrdU, Sigma Co., St. Lois, MO, USA) as a means to quantify the degree of tumor malignancy through DNA synthesis.

### Bioluminescence imaging *in vivo*

At week six, five mice from each group were anesthetized by isoflurane inhalation administered via the SBH Scientific anesthesia system (SBH Designs, Inc., Windsor, Ontario, Canada). Each anesthetized mouse received an intraperitoneal injection of 3 mg of D-luciferin potassium salts (Wako Pure Chemical Industries). Bioluminescence imaging with a Photon Imager (Biospace Lab, Paris, France) was performed. The bioluminescent signals received during the six minute acquisition time were quantified using Photovision software (Biospace Lab).

### Histopathological analyses

At necropsy following euthanasia at week six, the tumors and lymph nodes were removed from each mouse, fixed in 10% phosphate buffered formaldehyde solution and processed through to paraffin embedding. The lymph nodes from the axillary and femoral regions were routinely removed, along with lymph nodes that appeared abnormal. Other organs that appeared abnormal were also excised and preserved in the fixative solution. Lungs were inflated with formaldehyde solution prior to excision and immersion in fixative; the fixed individual lobes were subsequently removed from the bronchial tree and examined for metastatic foci and similarly processed through to paraffin embedding. All paraffin-embedded tissues were cut at 4 μm and sequential sections were either stained with hematoxylin and eosin (H&E) for histopathological examination or left unstained for immunohistochemical analysis.

### p53 and phospho-Akt immunohistochemistry

The labeled streptavidin-biotin (LSAB) method (Dako Co., Glostrup, Denmark) was used for p53 immunohistochemistry. Unstained sections were immersed in distilled water and heated for antigen retrieval prior to incubation with a p53 mouse monoclonal antibody (Clone Pab240, Santa Cruz Biotechnology) that reacts to the mutant protein in fixed specimens. Phosphorylated Akt expression in tissues was evaluated using phospho-Akt rabbit antibodies for Thr308 (Santa Cruz Biotechnology) and Ser473 (Cell Signaling Technology).

### Apoptosis and active-caspases in mammary tumors

For quantitative analysis of cell death, sections from paraffin-embedded tumors were assayed using the TUNEL method in conjunction with an apoptosis *in situ *detection kit (Wako Pure Chemical Industries), with minor modifications to the manufacturer's protocol. TUNEL-positive cells - regarded mainly as apoptotic cells - were counted in viable regions peripheral to areas of necrosis in tumor sections. The slides were scanned at low-power (× 100) magnification to identify those areas having the highest density of TUNEL-positive cells. Four fields neighboring an area of high TUNEL positivity were then selected and counted at higher (× 200-400) magnification. The number of TUNEL-positive cells was expressed as number per cm^2^.

Active caspase expression in the mammary tumor tissues was immunohistochemically detected using anti-cleaved caspase-3 and anti-cleaved caspase-9 rabbit polyclonal antibodies (Cell Signaling Technology). Immunohistochemistry was conducted using the LSAB method, and CSA II amplification (Dako) was additionally applied to detect cleaved caspase-9.

### DNA synthesis in mammary tumors

The tumors from five animals from each treatment group were subsequently evaluated for DNA synthesis rates as inferred by BrdU incorporation. DNA was denatured *in situ *by incubating unstained paraffin-embedded tissue sections in 4 N HCl solution for 20 minutes at 37°C. The incorporated BrdU was visualized after exposure to an anti-BrdU mouse monoclonal antibody (Clone Bu20a; Dako). The numbers of BrdU-positive S-phase cells per 250 mm^2 ^were counted in four random high power (× 400) fields of viable tissue and the BrdU labeling index was expressed as number per cm^2^.

### Blood microvascular density in mammary tumors

Immunohistochemistry based on the LSAB method (Dako) was performed to quantitatively assess blood microvessel density in primary mammary carcinomas; a rabbit polyclonal antibody against CD31 (Lab Vision Co., Fremont, CA, USA), a marker specific for blood vessel endothelium, was used. The numbers of CD31-positive blood microvessels were counted as previously described [27]; briefly, slides were scanned at low-power (× 100) magnification to identify those areas having the highest number of vessels and the five areas of highest microvascular density were selected and counted at higher (× 200-400) magnification.

### Dilated lymphatic vessels with cancer cell invasion

Mammary tumor sections from paraffin-embedded tissues were immunohistochemically stained using the LSAB method (Dako). A hamster anti-podoplanin monoclonal antibody (AngioBio Co., Del Mar, CA, USA), against a lymphatic endothelium marker was used. To quantitatively assess the number of lymphatic vessels having intraluminal tumor cells in the whole periphery area of the primary mammary carcinomas, the slides were scanned at low-power (x100) magnification to identify podoplanin-positive lymphatic vessels. Whether the lymphatic vessel contained mammary cancer cells or not was then confirmed at higher (x200-400) magnification [20].

### Statistical analysis

Significant differences in the quantitative data between groups were analyzed using the Student's *t*-test via the method of Welch, which provides for insufficient homogeneity of variance. The differences in metastatic incidence were examined by Fisher's exact probability test, with either *P *< 0.05 or *P *< 0.01 considered to represent a statistically significant difference. Survival rates were analyzed using the Holm-Sidak method.

## Results

### Cell viability of mammary carcinoma cells treated with α-mangostin

**Figure 2:**
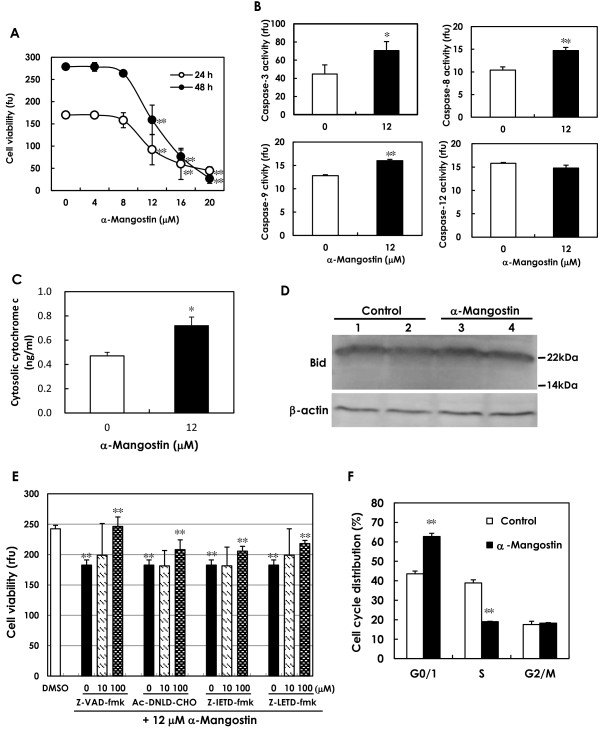
**Apoptosis and cell cycle *in vitro *study (mouse mammary carcinoma BJMC3879luc2 cells)**. **(A) **Cell viability was significantly lower in mouse mammary carcinoma BJMC3879luc2 cells treated with more than 12 μM α-mangostin for 24 or 48 hours (**P < 0.01). Five samples from each dosage of α-mangostin were examined. The IC_50 _concentration was determined to be 12 μM; therefore, 12 μM α-mangostin and 24 hour-incubation was used for all *in vitro *studies. (**B) **Caspase activities were evaluated using the luminescence assay. Activities of caspase-3, caspase-8 and caspase-9, but not caspase-12, were significantly elevated in BJMC3879luc2 cells treated with 12 μM α-mangostin for 24 hours (*P < 0.05 or **P < 0.01). Six samples from the control and eight samples from α-mangostin-treated cells were used for measurement of caspase-3 activity, and three samples from each group were used for activity measurements of the other caspases. (**C) **Cytochrome *c *in the cytosolic fraction, as determined by ELISA, was significantly increased in BJMC3879luc2 cells treated with α-mangostin for 24 hours compared to control levels (*P < 0.05). Three samples each from the control and α-mangostin-treated cell cultures were examined. (**D) **Western blots of Bid (22 kDa) in BJMC3879luc2 cells treated with or without α-mangostin for 24 hours were similar (upper panel). Cleaved Bid (15 kDa) was not observed after α-mangostin treatment. β-Actin served as the internal control (lower panel). (**E) **In BJMC3879luc2 cells treated with α-mangostin for 24 hours, cell viability was significantly increased by 10 or 100 μM of the broad-spectrum caspase inhibitor z-VAD-fmk, the caspase-3 specific inhibitor Ac-DNLD-CHO, the caspase-8 specific inhibitor z-IETD-fmk, and the caspase-9 specific inhibitor z-LETD-fmk (**P < 0.01). (**F) **Cell-cycle analysis showed that α-mangostin induced arrest in the G1-phase and inhibition of cells entering the S-phase in metastatic mouse mammary carcinoma BJMC3879luc2 cells (**P < 0.01). Data are presented as mean ± SD. Ac: acetyl; CHO: aldehyde; ELISA: enzyme-linked immunosorbent assay; fmk: fluoromethyl ketone; z: N-benzyloxycarbonyl.

Viability analyses of BJMC3879luc2 mammary cancer cells exposed to α-mangostin showed significantly decreased viability after 24 and 48 hours of treatment with > 12 μM compound (Figure 2A). Based on the IC_50 _data, 12 μM was determined to be the optimal concentration of α-mangostin for the *in vitro *studies.

### *In vitro *α-mangostin-induced apoptosis

#### Caspase activity

Significantly elevated caspase-3, caspase-8 and caspase-9 activity was observed in BJMC3879luc2 cells treated with α-mangostin for 24 hours (Figure 2B), compared to the respective controls. The activity of caspase-12 did not differ significantly between control cells and α-mangostin-treated cells (Figure 2B). Further, BJMC3879luc2 cells treated with 12 μM α-mangostin for 48 hours showed a greater number of apoptotic cells by TUNEL staining compared to the control (data not shown).

#### Release of cytochrome *c*

The levels of cytochrome *c *protein in cytosolic fractions were significantly higher in cells treated with α-mangostin alone for 24 hours (Figure 2C), strongly suggesting the engagement of the mitochondria-mediated apoptotic pathway.

#### Bid cleavage

Since caspase-8 activities were elevated, we examined whether the mitochondrial pathway via caspase-8-Bid cleavage occurred by performing western blots for Bid. Full-length Bid (22 kDa) was equally detected in control cells and in cells treated with α-mangostin only for 24 hour (Figure 2D). No cleaved Bid (15 kDa) was found in any of the groups.

#### Caspase inhibitor experiment

To determine whether caspase activation is necessary (possibly of caspase-independent apoptosis) to induce α-mangostin-induced apoptosis, a caspase inhibitor experiment was conducted. Inhibition of caspase activation through the application of all specific inhibitors resulted in protection of cell viability in α-mangostin-treated cultures when compared to cultures treated with α-mangostin alone (Figure 2E).

#### Cell cycle of mammary carcinoma cells treated with α-mangostin

As measured by flow cytometry, 24 hour exposure to 12 μM α-mangostin induced a significant elevation in the number of cells in the G1-phase compared with control cells (Figure 2F). There was also a significant reduction in the S-phase population in the α-mangostin-treated cell suspensions (Figure 2F).

#### *In vivo *survival rates, body weights and mammary tumor growth

**Figure 3:**
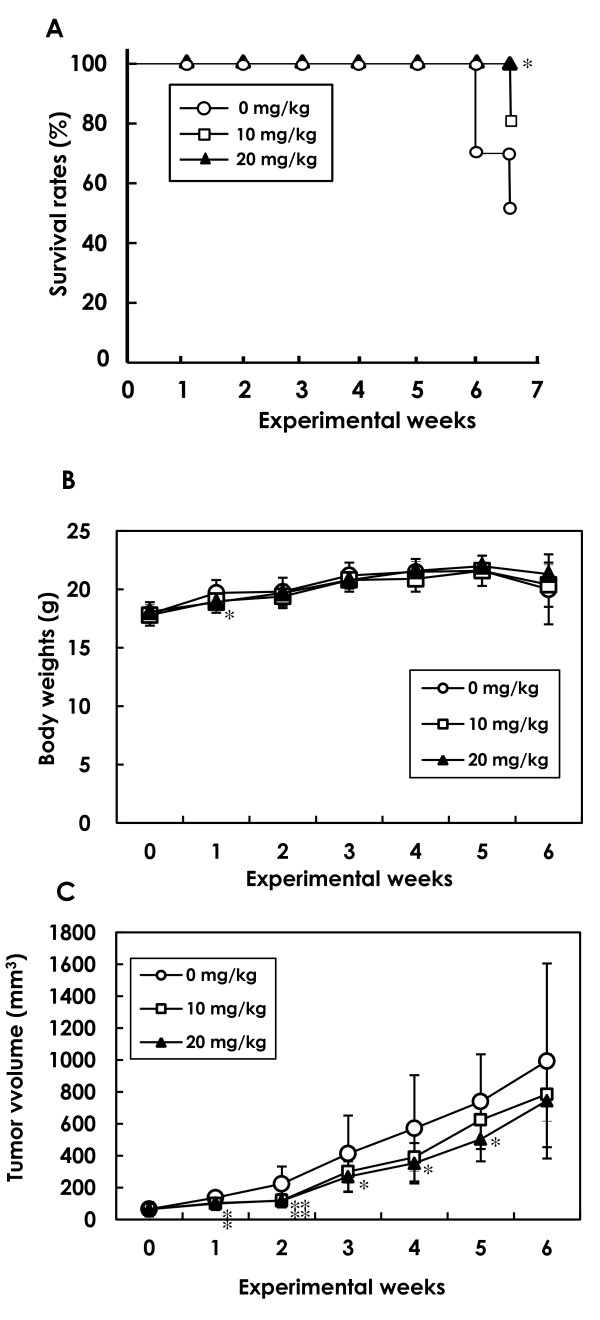
**Survival rates, body weights and tumor volumes in mammary carcinomas of mice treated with 0, 10 and 20 mg/kg/day α-mangostin**. α-Mangostin was administered via implanted mini-osmotic pumps. Each group consisted of ten mice. **(A) **Survival rates (%) were significantly greater in the 20 mg/kg/day group than the control group (*P < 0.05). **(B) **Body weights of mice treated with 10 and 20 mg/kg/day α-mangostin were similar to those in the control group with the exception of the 20 mg/kg/day mice at week one. **(C) **Increases in tumor volume in the 20 mg/kg/day group were significantly lower in weeks one to five compared to the control values (*P < 0.05; **P < 0.01). In the 10 mg/kg/day/day group, significant suppression of the tumor volume was observed in weeks one and two, with a decrease in growth suppression noted thereafter. Data are presented as mean ± SD.

Survival rates are shown in Figure 3A. Although five animals (50%) in the 0 mg/kg/day group and two animals (20%) in the 10 mg/kg/day/day group died by week 7 due to the widespread metastasis of mammary carcinoma, no mice died in the 20 mg/kg/day group, a statistically significant difference (*P *< 0.05). Body weight changes in control and α-mangostin-treated mice bearing mammary tumors are shown in Figure 3B. The weights of control and α-mangostin-treated mice bearing mammary tumors did not differ statistically throughout the experiment, with the exception of the 20 mg/kg/day mice at week 1.

Tumor volumes are presented in Figure 3C. Tumor growth, as inferred by computed volume, was significantly inhibited in the 10 mg/kg/day group from week 1 to 2 and in the 20 mg/kg/day group from week 1 to 5, compared with controls. By the end of the experiment, the average tumor volume in control animals was 993 ± 612 mm^3^, while the average tumor volume of mice that received 10 or 20 mg/kg/day α-mangostin was 785 ± 170 mm^3 ^and 744 ± 292 mm^3^, respectively.

### Mammary carcinoma metastasis with α-mangostin treatment

#### Bioluminescence imaging

**Figure 4:**
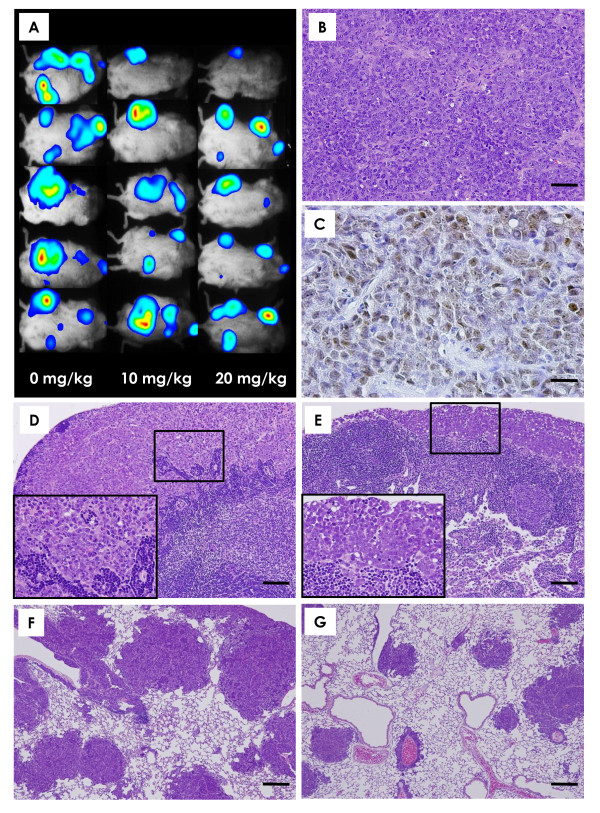
**Bioluminescent imaging and histopathological findings**. **(A) **Bioluminescent imaging in five representative mice from each group (0, 10 and 20 mg/kg/day groups). Bioluminescent imaging showed that the extension of metastasis tended to be lower in the α-mangostin-treated groups compared to the control group. **(B) **The implanted mammary carcinomas proved to be moderately differentiated adenocarcinoma. Scale bar = 50 μm. **(C) **p53 immunohistochemistry of mammary carcinomas induced by BJMC3879luc2 cell inoculation. Note the nuclear staining for abnormal p53 protein, indicating that these cells carry mutant p53. Scale bar = 25 μm. **(D) **Metastasis to a lymph node in a control mouse. Scale bar = 250 μm. Metastatic carcinoma cells filled the sinusoidal space (higher magnification in box, inset). **(E) **A lymph node from a mouse given 20 mg/kg/day α-mangostin. Scale bar = 250 μm. Metastatic carcinoma cells filled the subcapsular sinus (higher magnification in box, inset). **(F) **Metastatic foci in the lung of a control mouse. Many metastatic foci and small to large nodules were seen. Scale bar = 250 μm. **(G) **Metastatic foci in the lungs of mice given 20 mg/kg/day α-mangostin. Scale bar = 250 μm. **(A)**: *Bioluminescent imaging***; (B **and **D-G)**: *H&E stain*; **(C)**: *p53 immunohistochemistry*.

Bioluminescence imaging showed signal indicative of metastatic growth in the mandibular, axillary and inguinal lymphatic regions of all groups; however, there was a tendency for decreased metastatic expansion in mice treated with α-mangostin compared to control animals (Figure 4A). Histopathology of primary mammary tumors

Histopathologically, the mammary tumors induced by BJMC3879luc2 cell inoculation proved to be moderately differentiated adenocarcinomas (Figure 4B) containing mutated p53 as inferred by immunohistochemistry (Figure 4C).

### Lymph node metastasis

**Figure 5:**
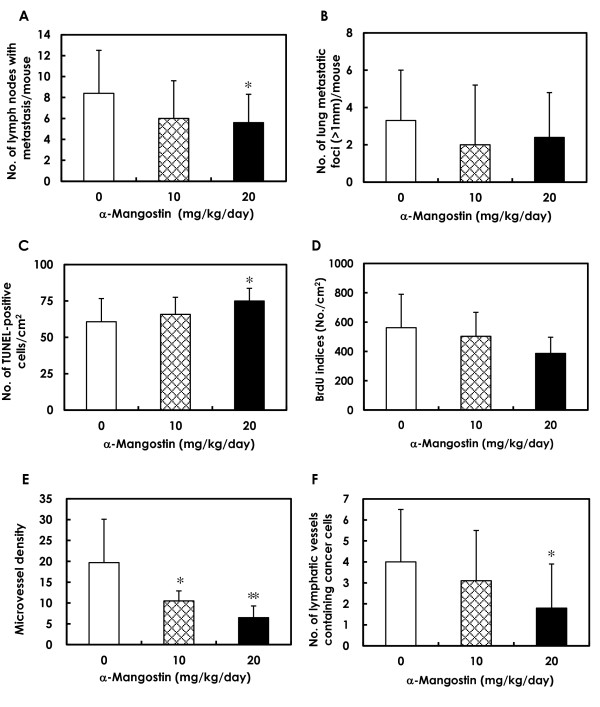
**Quantitative analyses of metastasis, apoptosis, DNA synthesis and vascular density in mammary carcinomas**. **(A) **The multiplicity of lymph node metastasis was significantly lower in the 20 mg/kg/day α-mangostin group (*P < 0.05). **(B) **The multiplicity of lung metastasis tended to be lower in the α-mangostin-treated groups, but this was not statistically significant. **(C) **Apoptotic cell death, assessed by TUNEL staining, was significantly higher in the 20 mg/kg/day α-mangostin group (*P < 0.05). **(D) **BrdU labeling indices tended to be lower in the 20 mg/kg/day α-mangostin group. **(E) **Microvessel density in tumors, inferred by CD31-positive endothelium, was significantly lower in the 10 and 20 mg/kg/day α-mangostin groups. **(F) **The number of dilated lymphatic vessels containing intraluminal tumor cells was significantly lower in the 20 mg/kg/day α-mangostin group than in the control group (*P < 0.05). Data are presented as mean ± SD. BrdU: 5-bromo-2'-deoxyuridine; TUNEL: terminal deoxynucleotidyl transferase-mediated dUTP-FITC nick end-labeling.

Representative examples of lymph node metastases are shown in Figures 4D and 4E. Lymph node metastasis occurred in 100% of mice in the 0 mg/kg/day/day group and in 90% of the mice in the 10 and 20 mg/kg/day groups. However, the number of metastasis-positive lymph nodes per mouse was significantly lower in the 20 mg/kg/day group compared to the control group (Figure 5A).

### Lung metastasis

Lung metastasis also occurred in all mice regardless of treatment (Figures 4F, G). Although the number of metastatic lung foci > 1 mm in diameter per mouse tended to be lower in the α-mangostin-treated groups, this decrease was not statistically significant (Figure 5B).

### *In vivo *apoptosis and DNA synthesis in mammary tumors of α-mangostin-treated mice

**Figure 6:**
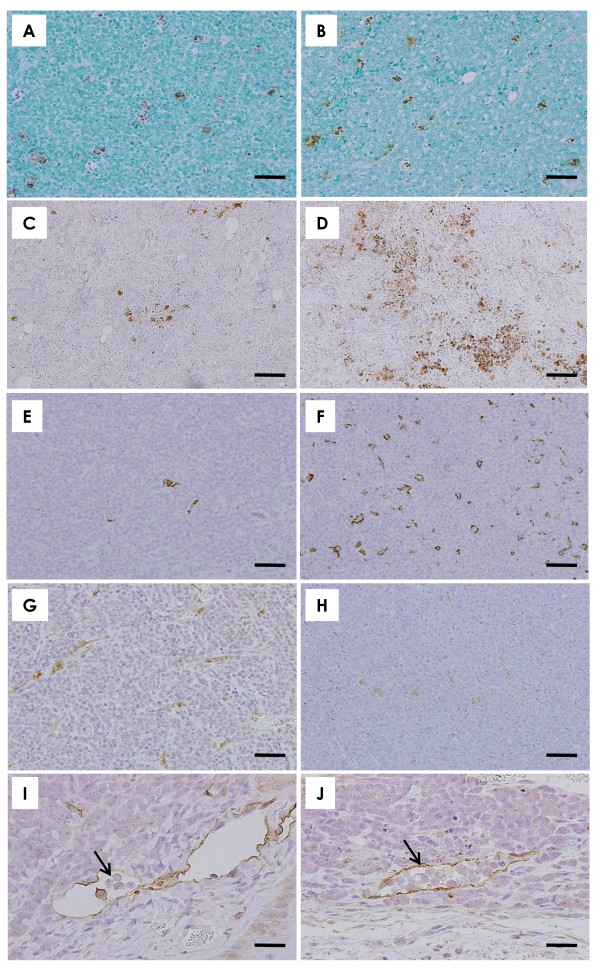
**Apoptosis, active-caspase expression, angiogenesis and dilated lymphatic vessels with cancer cell invasion in mammary carcinomas**. TUNEL-positive apoptotic cells are much more frequently seen in the tumor of a mouse given 20 mg/kg/day α-mangostin **(B) **than in the tumor of a control mouse **(A)**. Expression of active caspase-3 **(C, D) **and active caspase-9 **(E, F) **was more prominent in the tumor of a mouse given 20 mg/kg/day α-mangostin **(D, F) **than in a control mouse **(C, E)**. The number of CD-31-positive endothelial cells was lower in the tumor of a mouse given 20 mg/kg/day α-mangostin **(H) **compared to that of a control mouse **(G)**. Podoplanin-positive lymphatic vessels of a tumor in a control mouse were often dilated and filled with tumor cells **(**arrow, **I)**. Mammary cancer cells were also observed in the intraluminal space of the dilated lymphatic vessels in the tumor of a mouse given 20 mg/kg/day α-mangostin **(**arrow, **J)**. **(A-H)**: Scale bar = 50 μm; **(I, J)**: Scale bar = 25 μm. **(A, B)**: *TUNEL stain; ***(C, D)**: *active caspase-3 immunohistochemistry*; **(E, F)**: *active caspase-9 immunohistochemistry*; **(G, H)**: *CD31 immunohistochemistry*; **(I, J)**: *podoplanin immunohistochemistry*.

Representative examples of TUNEL-positive cells from mammary tumor tissues are presented in Figures 6A and 6B. The results of the quantitative analysis for apoptosis in mammary tumors, as assessed by the TUNEL assay, are shown in Figure 5C. The number of TUNEL-positive cells was significantly higher in tumors from the 20 mg/kg/day groups as compared to the numbers in tumors from the control mice (Figure 5C). Immunohistochemistry demonstrated much higher expression of the active forms of caspase-3 and caspase-9 in mammary tumors of mice treated with α-mangostin (Figures 6D, F) when compared to those of the control animals (Figures 6C, Figure 6E), suggesting that α-mangostin induces mitochondria-mediated apoptosis in mammary tumor cells *in vivo *as well as *in vitro*.

DNA synthesis levels in mammary carcinomas of α-mangostin-treated mice, as inferred by BrdU labeling indices, are shown in Figure 5D. The level of DNA synthesis in tumors tended to be lower in the 20 mg/kg/day group, but this decrease was not statistically significant (Figure 5D).

### Effect of α-mangostin on microvascular density and cancer cell migration via lymphatic vessels

Microvessel density, as determined by immunohistochemical analysis with the blood vessel endothelial cell marker CD31 (Figures 6G, H), was significantly lower in the 10 and 20 mg/kg/day α-mangostin groups compared to the 0 mg/kg/day group (Figure 5E).

The lymphatic vessels in mammary tumors were stained with anti-podoplanin antibody, as demonstrated in Figures 6I and 6J, to detect malignant cell migration. Tumor cells were found within the lumina of dilated lymphatic vessels of tumors in both the control (Figure 6I) and α-mangostin-treated (Figure 6J) animals; however, the number of dilated lymphatic vessels containing intraluminal tumor cells (arrows in Figures 6I, J) was significantly lower in mammary tumors of mice given 20 mg/kg/day of α-mangostin (Figure 5F), indicating a reduction in the number of tumor cells migrating into the lymphatic vessels of tumor tissues.

### Effects of α-mangostin on Akt phosphorylation

#### *In vitro*

**Figure 7:**
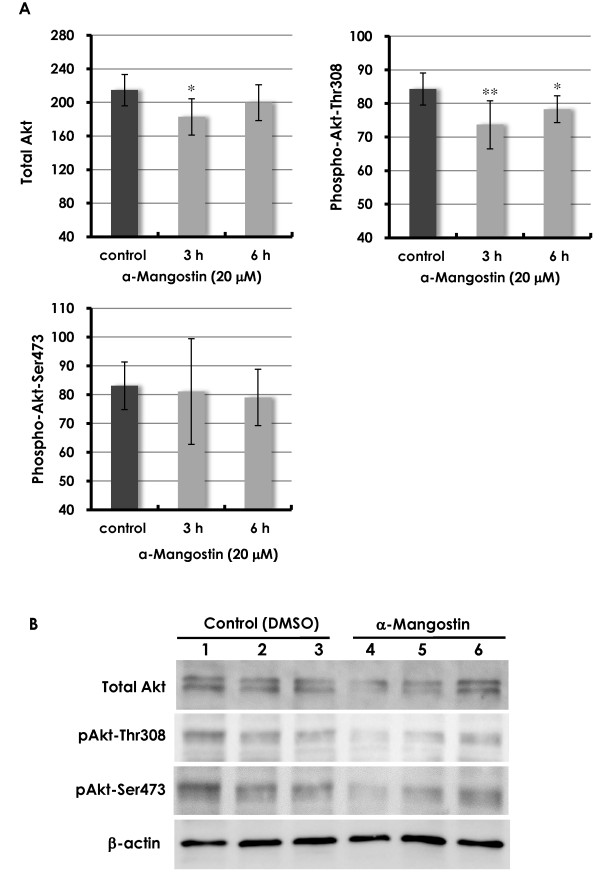
**Akt phosphorylation *in vitro***. Akt phosphorylation plays a central role in a variety of oncogenic processes. **(A) **Quantitative analysis of phospho-Akt in MDA-MB231 cells treated with 20 μM α-mangostin showed that although total Akt showed a transient decease at 3 hour, the levels returned to the control levels. The levels of phospho-Akt-Thr308 were significantly lower at 3 hour and 6 hour, but this was not seen for phospho-Akt-Ser473, which showed large variations. **(B) **Western blotting showed that there were no apparent differences between control and α-mangostin-treated cells in total Akt; however, phospho-Akt-Thr308 levels tended to be lower in α-mangostin-treated cells. Phospho-Akt-Ser473 showed a tendency to be slightly lower in α-mangostin-treated cells in western blotting. Ser473: serine 473; Thr308: threonine 308

As shown in Figure 7A, the levels of total Akt in α-mangostin-treated cells had transiently decreased at 3 hours, but had recovered by 6 hours. Although the levels of phospho-Akt-Thr308 in α-mangostin-treated cells were significantly lower at 3 hours and 6 hours, the levels of phospho-Akt-Ser473 were unchanged but with large variations at 3 hours. Western blots revealed that, although there were no apparent differences in total Akt levels between control and α-mangostin-treated mammary carcinoma cells, phospho-Akt-Thr308 was lower in mammary carcinoma cells treated with α-mangostin for 24 hours (Figure 7B). There were wide variations among mice, but, overall, phospho-Akt-Ser473 tended to marginally decrease with α-mangostin treatment (Figure 7B).

#### *In vivo*

**Figure 8:**
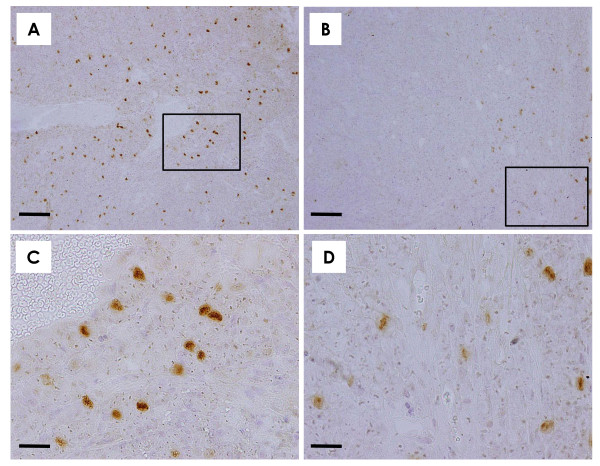
**Akt phosphorylation *in vivo***. Akt phosphorylation plays a central role in a variety of oncogenic processes. Compared to the number of cells positive for phospho-Akt-Thr308 in the mammary carcinoma tissue of a control mouse **(A)**, the number of phospho-Akt-Thr308-positive cells was markedly lower in the mammary carcinoma tissue of a mouse treated with 20 mg/kg/day α-mangostin **(B)**. Note that stronger nuclear and weaker cytoplasmic staining was observed in both a control mouse carcinoma **(C**, higher magnification of the box in **A) **and a 20 mg/kg/day α-mangostin-treated mouse carcinoma **(D**, higher magnification of a box in **B)**. The numbers and intensities of stained cells were weaker in the 20 mg/kg/day group **(B, D**) than in the control group **(A, C)**. **(A**, **B)**: Scale bar = 100 μm; **(C, D)**: Scale bar = 25 μm. **(A-D)**: *Phospho-Akt-Thr308 immunohistochemistry*.

Compared to expression of phospho-Akt-Thr308 in control mammary carcinomas (Figure 8A), the number of positive cells and their staining intensity was markedly lower in mammary carcinomas of mice treated with 20 mg/kg/day α-mangostin (Figure 8B). As shown under higher magnification, strong nuclear expression with weaker cytoplasmic expression of phospho-Akt-Thr308 was observed in mammary carcinoma cells in the control (Figure 8C) and 20 mg/kg/day groups (Figure 8D). However, the levels of phospho-Akt-Thr308 were much lower in the 20 mg/kg/day group than in the control group. There was no apparent difference between phospho-Akt-Ser473 expression between the control and α-mangostin 20 mg/kg/day groups (data not shown).

## Discussion

The present study showed that treatment with 20 mg/kg/day α-mangostin resulted in prolonged survival rates and increased inhibition of tumor growth and lymph node metastasis in an immunocompetent mouse metastatic mammary carcinoma model containing a p53 mutation. Mammary carcinoma tissues of mice treated with 20 mg/kg/day α-mangostin showed elevation of apoptotic cell death, increased expression of active caspase-3 and -9, and a decrease in the number of cells with phospho-Akt-Thr308. Furthermore, decreased blood microvessel density and fewer numbers of lymphatic vessels containing intraluminal cancer cells were observed in mammary carcinomas of α-mangostin-treated mice. Our *in vitro *studies have demonstrated that α-mangostin induces mitochondria-mediated apoptosis, but not via the Bid-mitochondria cross-talk pathway, G1-phase arrest, or S-phase suppression in the cell cycle.

Akt phosphorylation contributes to cell proliferation, anti-apoptotic cell death, cell cycle entry, angiogenesis and metastasis - all important aspects of the oncogenic process [28]. The phosphoinositide 3-kinase (PI3K)/Akt pathway is now considered to be an important therapeutic target for cancer. Indeed, Akt inhibitors have shown significant promise preclinically and are now in clinical trials [29]. Full activation of Akt is a multistep process, and the final step is phosphorylation of Akt1 at two sites, Thr308 and Ser473 [30]. Upon activation, Akt moves to the cytoplasm and nucleus where it phosphorylates downstream target proteins. We demonstrated that treatment with α-mangostin decreased phospho-Akt-Th308. In this study, although intense staining of phospho-Akt-Th308 in both the cytoplasm and nucleus was immunohistochemically observed in mammary carcinoma tissues of the control group, the intensity and number of cells expressing phospho-Akt-Th308 tended to be lower in the 20 mg/kg/day α-mangostin group. Since both Thr308 and Ser473 are necessary for full activation of Akt [30,31], the fact that α-mangostin reduced phospho-Akt-Th308 *in vitro *and *in vivo *suggests downstream inhibition of pathways in Akt. Several modes of Akt pathway dysregulation have been identified in various types of cancer, including breast cancer, and this ultimately affects a number of processes including cell growth, survival, proliferation, and motility and/or invasion [28]. Therefore, the observation in the present study of reduced tumor growth, apoptotic cell death, cell-cycle alterations, anti-angiogenesis and anti-metastasis may be partially responsible for inhibition of Akt phosphorylation.

As previously stated, we demonstrated a significant induction of apoptosis with α-mangostin in murine mammary carcinoma cells both *in vitro *and *in vivo*. There are two pathways currently proposed to play major roles in regulating apoptosis in mammalian cells: a pathway mediated by the death receptor (an extrinsic pathway, executed by caspase-8), and a pathway mediated by mitochondria (intrinsic pathway with execution by caspase-9) [32]. In addition, however, endoplasmic reticulum (ER) stress has been shown to switch the signaling direction from the pro-survival to the pro-apoptotic pathway [33]. Caspase-12, a caspase localized in the ER, is known to mediate this switch in mice [34]. Caspase-3 is the final executor of apoptosis. Many of the apoptotic signals are transduced to the mitochondria, decreasing the mitochondrial membrane potential and leading to the release of cytochrome *c *from the mitochondrial lumen into the cytoplasm. The released cytochrome *c *binds to the apoptosis protease-activating factor-1 (Apaf-1), and this complex activates caspase-9. Caspase-8 also has a cross-talk pathway to the mitochondrial pathway through the cleavage of Bid [32].

*In vitro*, we noted increased activity of caspases-3, -8 and -9 and increased cytosolic cytochrome *c *levels in α-mangostin-treated mammary carcinoma cells, suggesting that α-mangostin at least initiated mitochondria-mediated apoptosis. In fact, mammary carcinoma tissues of α-mangostin-treated mice showed strong expression of active caspase-3 and -9, demonstrating that mitochondria-mediated apoptosis actually occurred *in vivo *as well. All caspase inhibitors, including that for caspase-8, completely rescued α-mangostin-induced cell death in cultures. Bid cleavage, however, was not observed, indicating that cross-talk between caspase-8 and Bid may not be involved here. The question arises as to why caspase-8 activity nevertheless increased. Caspase-8 participates in ERK activation, and this participation is attributed to the Death Effector Domains (DED) of caspase-8 [35] and a direct association between ERK and a DED-containing fragment of caspase-8, with co-transport of an ERK-caspase-8-DED complex to the nucleus during apoptosis, has been reported [36]. The caspase-8-ERK pathway may also play a role in α-mangostin-induced apoptosis, but further investigation is required to elucidate this mechanism. Since no elevation in caspase-12 activity was seen in the present study, α-mangostin-induced apoptosis may not have involved ER stress. Our current experiments suggest that α-mangostin-induced apoptosis in BJMC3879luc2 cells, which contain a p53 mutation, occurs through a p53-independent mechanism.

The tumor suppressor gene *p53 *encodes a transcription factor that plays a critical role in regulating cell cycle progression, DNA repair, and cell death. *p53 *is the most frequently altered gene in human cancers and loss of functional p53 protein occurs in a majority of epithelial ovarian cancers. The present experiments suggest that α-mangostin-induced apoptosis in BJMC3879luc2 cells having a *p53 *mutation occurs through a p53-independent mechanism. Since 50% of human cancers have *p53 *mutations [37], the fact that the α-mangostin induces a p53-independent apoptotic response in cancer cells having a *p53 *mutation may be highly relevant to inhibiting many human cancers. In the case of non-functional p53 status, p73, the p53 homologue, may play a role in apoptosis induction. On a related note, it has been shown that stroma-specific loss of heterozygosity or allelic imbalance is associated with *p53 *mutations and regional lymph-node metastases in sporadic breast cancer [38].

Neovascularization is a key process in the growth of solid tumors, and tumors will not grow beyond a few cubic millimeters unless a vascular network is established to feed further expansion [39]. In the present study, we demonstrated that treatment with α-mangostin significantly reduced microvessel density in mammary carcinomas. We have also recently shown that panaxanthone, which is comprised of approximately 75% to 85% α-mangostin and 5% to 15% γ-mangostin with the sum of both contents > 90%, also inhibits tumor growth and metastasis in mouse mammary carcinomas and is also associated with decreased tumor angiogenesis. Since the growth of both primary tumors and of metastases is angiogenesis-dependent, and since microvascular endothelial cells recruited by a tumor in the process of neovascularization have become an important second target of cancer therapy [40], the anti-angiogenic properties of α-mangostin may be very important to the development of cancer therapies, particularly those involved with molecular targeting in neoplasms.

Tumor cell dissemination is mediated by a number of mechanisms, including direct invasion into local tissue, lymphatic spread, and hematogenous spread. In general, the most common pathway of initial dissemination is via the lymphatics, with patterns of spread via afferent ducts [41]. The lymphatic capillaries present in tissues and tumors provide entrance into the lymphatic system, allowing cancer cell migration to the lymph nodes. Breast cancer cells are known to disseminate through the body by all of the above mechanisms; common metastatic sites are the lymph nodes, lung, bones, and liver [5]. Lymph node involvement remains specifically the most influential prognostic factor in breast cancer progression [42]. In the present study, the multiplicity of lymph node metastases was decreased in α-mangostin-treated mice. This phenomenon was supported by a significant decrease in the number of lymphatic vessels demonstrating intraluminal tumor cells in the α-mangostin-treated groups. This indicates that α-mangostin has an inhibitory effect on migration into lymphatic vessels. Other investigators have reported that α-mangostin exerts inhibitory effects on cell invasion and migration in mammary cancer cells due to downregulation of MMP-2 and MMP-9 [12], and we cannot preclude that this mechanism was not operating in our study, as well.

Significant elevations of NK activity were recently reported in mice treated with crude α-mangostin at 20 and 40 mg/kg/day compared to control mice [25]. In a human pilot study, healthy people orally administered panaxanthone, a less purified α-mangostin analog, at a dose of 150 mg/day/person for seven days also showed significant increases in NK activity [25]. Since the mammary cancer model used in the current study was an immunocompetent model, elevation in NK activity would be expected in the 20 mg/kg/day α-mangostin group.

The PI3K pathway exerts its regulatory functions on cell proliferation, cell transformation, cell apoptosis, tumor growth and angiogenesis through downstream targeting of Akt [43]. Expression of Akt in a dominant/negative mutant also inhibited angiogenesis and tumor growth, and also decreased the expression of HIF-1α and vascular endothelial growth factor (VEGF) in tumor xenographs [44]. Akt has also been reported to phosphorylate and activate endothelial nitric oxide synthase (eNOS), which contributes to angiogenesis through endothelial nitric oxide production. Activation of Akt by VEGF orchestrates several signaling events that contribute to angiogenesis [45]. Involvement of PI3K, Akt and eNOS in endothelial cell biology is apparent under both physiological and pathological conditions. *Akt1 *null mice show reduction of lymphatic capillary vessel size as well as defects of smooth muscle cell coverage and valve development, suggesting that Akt1 is a required isoform in lymphangiogenesis [46]. Thus, Akt-mediated signaling plays an important role in lymphangiogeneis as well as in angiogenesis. Since α-mangostin reduced Akt phosphorylation *in vitro *and *in vivo *in the present study, this signaling may be responsible for reduction of vasculogenesis in our mouse tumors.

Studies have also shown that α-mangostin decreases the levels of cyclooxygenase-2 (COX-2) and inducible nitric oxide synthase in macrophages [47]. COX is a key enzyme that catalyzes the conversion of arachidonic acid to prostaglandin E_2 _(PGE_2_). Recent studies have shown that overexpression of COX-2 and PGE_2 _is a characteristic of many human cancers [48-50] and selective COX-2 inhibitors have shown significant effects in reducing the incidence and progression of tumors and metastasis in animal models of mammary cancer [51-53]. Therefore, the observed antitumor action by α-mangostin in our present study may be due to reduction of COX-2 levels. Akt signaling has been reported to upregulate COX-2 expression through the NF-*k*B/I*k*B pathway in mutated PTEN endometrial carcinoma cells [54]; α-mangostin may be able to reduce COX-2 expression through Akt dephosphorylation.

Estrogen and the estrogen receptor α (ERα) are widely recognized to play a crucial role in the development and progression of hormone-dependent breast cancer. The BJMC3879luc2 mammary carcinoma cells used in the present study have been previously characterized as having cytoplasmic location of ERα and a partial weak response to estrogen treatment [17]. When BJMC3879luc2 cells are implanted into mice, ERα mRNA levels rise significantly higher in females than males. Raloxifene, a selective estrogen receptor modulator, inhibits tumor growth and metastasis in the same mouse metastatic mammary carcinoma model as we used in the present study [17]. Aromatase is an estrogen synthase responsible for catalyzing the biosynthesis of estrogens from androgens. Since both α- and γ-mangostin inhibit aromatase activity in a dose-dependent manner, this is another possible mechanism by which α-mangostin exhibited the antitumor effect seen in the present study.

The population is aging in many modern societies, and since the morbidity rates of cancer and cerebrovascular disease are increasing steadily, preventive medicine, in addition to therapeutic treatments, is becoming increasingly important. Many medically advanced societies are exploring both Western medicine and Oriental alternatives, and the demand for complementary and alternative medicines is on the rise. In fact, the 2007 National Health Interview Survey by the National Center for Complementary and Alternative Medicine (NCCAM) and the National Center for Health Statistics showed that 38% of adults in the United States are using some form of complementary and alternative medicine [55]. However, scientific data based on the principle of evidence-based analysis is still scant in the fields of complementary and alternative medicine. α-Mangostin, isolated from the pericarp of the mangosteen fruit, has been shown to induce many biological actions, such as anti-bacterial activity [56], apoptosis [7-10], and cell-cycle arrest [7]. An analog, panaxanthone (approximately 75% to 85% α-mangostin and 5% to 15% γ-mangostin with the sum of both contents > 90%) has been shown to significantly suppress tumor growth and metastasis in a mouse model of mammary cancer when administered in the diet [14]. These and other basic investigations provide a scientific basis for the anecdotal effects of α-mangostin.

## Conclusions

We have demonstrated that treatment with α-mangostin induces a significant increase in survival and suppression of tumor growth and lymph node metastasis in a mouse mammary cancer model carrying a p53 mutation. Given that lymph node involvement is the most important prognostic factor in breast cancer patients, the antimetastatic activity of α-mangostin, in particular, may be a crucial finding with future clinical application. In addition, α-mangostin may be useful as an adjuvant therapy or complementary alternative medicine and, possibly, as a tool for the chemoprevention of breast cancer development.

## List of abbreviations

Apaf-1: apoptosis protease-activating factor-1; BrdU: 5-bromo-2'-deoxyuridine; CHO: aldehyde; COX-2: cyclooxygenase-2; DED: death effector domain; DMSO: dimethyl sulphoxide; eNOS: endothelial nitric oxide synthase; ER: endoplasmic reticulum; ERα: estrogen receptor α; fmk: fluoromethyl ketone; GAPDH: glyceraldehyde-3-phosphate dehydrogenase; GFP: green fluorescence protein; H&E: hematoxylin and eosin; HRP: horseradish peroxidase; LSAB: labeled streptavidin-biotin; NCCAM: National Center for Complementary and Alternative Medicine; PI3K: phosphoinositide 3-kinase; Ser473: serine 473; Thr308: threonine 308; TUNEL: terminal deoxynucleotidyl transferase-mediated dUTP-FITC nick end-labeling; VEGF: vascular endothelial growth factor; z: N-benzyloxycarbonyl.

## Competing interests

This investigation was partially supported by a grant from PM Riken-yakka Ltd. and Field & Device Co. but the authors declare that they have no competing interests.

## Authors' contributions

MAS carried out all animal experiments as well as the histopathological analysis and performance of Western blots for Akt phosphorylation. Extraction of α-mangostin from mangosteen pericarps was performed by MI. Maintenance of mammary carcinoma cells and transplantation was performed by JM. Western blots for Bid and cell-cycle analyses were performed by HK. Quantitative analysis of Akt *in vitro *was performed by KA and YO (Kyoto University). MAS participated in the design of the study and wrote the first version of the manuscript. All authors have read and approved the final submitted manuscript.

## Pre-publication history

The pre-publication history for this paper can be accessed here:

http://www.biomedcentral.com/1741-7015/9/69/prepub
